# Ethnicity, gender, and social class in Honduras: an analysis using the permanent household survey

**DOI:** 10.3389/fsoc.2026.1758677

**Published:** 2026-05-26

**Authors:** Sara Ney Simons, David Pineda Talavera, José Octavio Llopis Hernández, Kevin Alberto Cruz, Mario Enrique Pineda Talavera, Cristian Sierra Cáceres, Delmer Roberto Marcía Hernández

**Affiliations:** 1 Department of Sociology, Faculty of Social Sciences, National Autonomous University of Honduras, Tegucigalpa, Honduras; 2 Department of Social Work, Universidade Federal de Vicosa, Viçosa, Brazil

**Keywords:** Afro-descendants, EGP classification, gender, Honduras, indigenous peoples, labor market, social stratification

## Abstract

**Introduction:**

Social stratification in Honduras is shaped by persistent informality, territorial inequality, and the intersection of ethnicity, gender, education, and rurality. Despite growing interest in intersectional approaches to class analysis in Latin America, Honduras remains underexplored in international stratification research.

**Methods:**

Using data from the 2024 Permanent Household Survey and an adaptation of the Erikson–Goldthorpe–Portocarrero (EGP) class scheme, we analyze the class positions of indigenous, Afro-descendant, and mestizo populations in Honduras, examining how ethnicity, gender, educational attainment, and rural–urban location interact to shape labor market outcomes.

**Results:**

Ethnic differences in class position are strongly moderated by education and geographic location: low educational attainment and rural residence increase the probability of insertion into agricultural or informal classes, while urban environments and higher education facilitate access to service-sector and formal employment. Women—particularly Afro-descendant and indigenous women—are concentrated in urban self-employment and service activities despite reaching educational levels comparable to men, with limited access to formal and protected occupations. Afro-Honduran women tend toward non-manual or autonomous urban work, while indigenous women are more concentrated in agricultural, domestic, or subsistence activities, often underreported in labor statistics.

**Discussion:**

The results demonstrate that class structure in Honduras operates intersectionally: no single factor—ethnicity, gender, education, or territory—explains class belonging independently, as their effects are mutually conditioned. Rurality functions not merely as a geographic dimension but as a structural axis of inequality, perpetuating historical disadvantages through limited productive diversification and scarce formal employment. These findings suggest that policies addressing education, employment, gender, or ethnicity in isolation are insufficient to alter existing class hierarchies, and call for integrated interventions that simultaneously tackle labor segmentation, educational inequality, territorial disparities, and racial and gender discrimination.

## 1 Introduction

Social inequality in Latin America is a long-term structural characteristic, associated with persistent class hierarchies and limited intergenerational mobility (World Bank, 2024; Solís and Boado, 2016). Although progress has been made in recent decades in reducing poverty and expanding education, these processes have not substantially altered the class structure or the weight of social origin in labor trajectories, even among cohorts with a higher level of education (Zenteno and Solís, 2006; Solís et al., 2019). In this context, social stratification continues to be multidimensional and is organized around education, occupational position, access to cultural resources, and segmentation between formal and informal employment (Solís and Boado, 2016).

Regional literature consistently demonstrates the intertwining of these class hierarchies with gender and ethnicity inequalities. Labor participation gaps between men and women persist at all levels of education and are closely linked to the unequal distribution of unpaid care work, which limits women’s economic autonomy (ILO, 2024). According to Martínez-Martínez et al. (2024), recent phenomena such as the COVID-19 pandemic widened the structural gaps that already existed, producing unequal impacts that most strongly harmed women and those who work in informal jobs in the region.

Similarly, indigenous and Afro-descendant populations face structural disadvantages in income and job opportunities, even when they reach higher levels of education, as a result of occupational segmentation, territorial barriers, and racialized discrimination (World Bank, 2015; Telles and Martínez Casas, 2019; Bento et al., 2019).

In this regional scenario, Honduras is a particularly relevant empirical case for analyzing the articulation between class, gender, and ethnicity. The country combines high levels of labor informality, a marked concentration of income, and an institutional historical trajectory that has made its ethnic diversity invisible in the construction of the nation-state (Llopis et al., 2024; Palacios, 2007). Although Honduras is home to nine indigenous and Afro-Honduran peoples (Lenca, Chortí, Nahual, Tolupan, Pech, Tawahka, Miskitu, Garifuna, and English-speaking blacks (Creole)) this heterogeneity has been systematically underestimated in official discourses and statistical production, limiting its incorporation into analyses of social stratification.

According to Euraque (2004), mixing in Honduras functioned as a mechanism of symbolic integration and material exclusion, since it incorporated indigenous peoples into the national identity but relegated them to economic development. Euraque (2023) also argues that the State constructed differences as invisibility and indifference, as normality. In addition, Rivas (1994) shows historical evidence that indicates that the conquest represented for the native peoples the dispossession of their territories and the beginning of a long political and economic subordination. These historical events not only created a system of political and land control but also set up ongoing inequalities that still affect the job opportunities and social status of indigenous and Afro-descendant people in today’s Honduras.

Moreover, we cannot understand the configuration of the class structure in Latin America without considering the historical exclusions that defined gender and citizenship. The Spanish-American constitutions of the nineteenth century constructed an implicitly male political citizenship, excluding women from decision-making and representation spaces due to cultural belief in their “*natural incapacity*.” Villars (2006, pp. 293, 299) demonstrates how gender determines who can obtain political recognition and access to economic and educational resources that influence class positions and structure social hierarchies.

Most of the research that analyzes the social structure in Latin America has used the framework established by Erikson et al. (1979), (EGP, hereinafter). They describe classes as relational positions characterized by labor connection, work regulation, and corresponding risks and protections. Rose and Harrison (2007) argue that modifying the model requires recognizing differences in employment systems and ethnic-racial labor divisions. Solís and Boado (2016) and Zenteno and Solís (2006) argue that, in a region characterized by high levels of informality, significant self-employment, and occupational segmentation, the traditional classifications of the EGP model require adjustments to accurately represent these characteristics. Each nation must incorporate gender and ethnicity into its analysis.

Although research on mobility and social stratification in Latin America has advanced significantly, the representation of Central America remains limited in the comparative literature (Solís and Boado, 2016; Solís et al., 2019). Most studies have focused on countries with more formalized labor markets, where mobility is analyzed based on protected occupations, leaving behind contexts characterized by high levels of informality and ethnic-racial segmentation, such as those in Central America. This omission hinders the regional understanding of contemporary stratification mechanisms by making intersectoral inequalities in societies with high structural heterogeneity empirically invisible.

In the case of Honduras, studies on social stratification and ethnicity are scarce and have been developed mainly from historical and anthropological approaches (Rivas, 1994; Palacios, 2007; Euraque, 2004, 2023). While these contributions are essential for comprehending the dynamics of racialization and exclusion, there is a lack of quantitative analyses that systematically investigate the interplay between social class, gender, and ethnic-racial self-identification in the labor market. Recent efforts to characterize the class structure in Honduras (Llopis et al., 2024) open up a still incipient field of research, in which the need to integrate these dimensions together persists.

Studies of social stratification can make both empirical and analytical progress by incorporating the Honduran case. From an empirical perspective, the analysis of a context characterized by labor informality and ethnic diversity contributes to remedying Central American underrepresentation in the regional literature. From an analytical perspective, the application of the Erikson–Goldthorpe–Portocarrero (EGP) scheme adapted to Latin America offers the possibility of examining how social class, gender, and ethnicity are articulated in the configuration of job opportunities in a context little explored by comparative studies.

To address this objective, the study uses a quantitative design of correlational scope based on the Permanent Multipurpose Household Survey (EPHPM) of Honduras for the year 2024. Based on a national sample of 41,129 employed people between 18 and 65 years of age, multinomial logistic regression models are estimated to analyze the probability of belonging to different social class positions (defined by an EGP scheme adapted to the Honduran context) according to ethnic-racial self-identification, sex, educational level, and geographic location. This approach allows us to identify patterns of intersectional stratification in a labor market characterized by high informality and productive heterogeneity, providing relevant empirical evidence to understand how these dynamics operate in Honduras and, by extension, in other Central American contexts with similar labor structures.

## 2 Methods

The main objective of this study was to analyze the association between ethnicity, gender, and position within the social class structure. The study uses a quantitative, correlational design. The source of information was the Permanent Multipurpose Household Survey (EPHPM) of the National Institute of Statistics for the year 2024. This survey used a two-stage, stratified, and probabilistic sampling method.

The EPHPM provides official social and economic indicators and includes a complex sampling design and weighting system. In general surveys, the estimate is structured by 4 domains: Central District, San Pedro Sula, urban, and rural. Each area is given a set “basic expansion factor,” and this factor is adjusted based on how many segments are surveyed and the number of homes in each segment (usually six), leading to preliminary counts that specialists then refine to create the final expansion factor, which takes into account the specific urban and rural differences in the country. This study uses the EPHM that consolidates all the surveys carried out during 2024, which contains a departmental weighting. In this case, the process follows the same method, but instead of using 4 areas, it adjusts for 18 departments, which includes all the political-administrative divisions of Honduras; the final expansion factor is calculated for each department and connected to population estimates, ensuring consistency between sample estimates and official population projections.

The total sample for the reference year consisted of 71,661 individuals nationwide, of which 52.7% resided in urban areas and 47.3% in rural areas. This study restricted the sample size to individuals aged 18 to 65 years, resulting in 41,129 participants within this age range.

The methodological strategy included two stages. First, a description of the population studied was made according to demographic characteristics such as sex, geographical area, and educational level. Second, two multinomial logistic regressions were estimated to calculate the probability of belonging to a social class according to the reference ethnic group. The first regression was estimated with a vector of regressors without interaction; the second model included an interaction term between ethnicity and sex, Table 1 describes the variables used.

**Table 1:** Analysis variables.

Dependent variable	
Social class	Class I. Class of Services
	Class II. Routine Formal Workers
	Class III. Small employers and freelancers
	Class IV. Salaried formal workers
	Class V. Salaried and self-employed workers in the informal sector
	Class VI. Independent agricultural class
	Class VII. Salaried agricultural class

Independent variables	
Ethnic group	Afro-Hondurans
	Indigenous peoples
	Mestizos
Sex	Male
	Female
Geographical area	Urban
	Rural
Educational level	No formal education
	Primary
	Secondary
	Superior

Source: authors.

The dependent variable “social class” was created using the Erikson, Goldthorpe, and Portocarrero (EGP) model, with changes made for Latin America to reflect the unique features of the local situation, especially the diverse and informal job markets (Solís et al., 2016; Chávez Molina et al., 2022). The EGP model and the European Socioeconomic Classification (ESeC) have documented measurement limitations, particularly regarding occupational coding and employment relations (Rose and Harrison, 2010) emphasize that the validity of the schema depends on how the occupational classification and coding are constructed; the criteria used to identify direct supervision; the difficulties in assigning the same occupation to different employment relationships depending on the sector and the type of contractual relationship; how autonomy and skills are operationalized; the conditions of societies with high levels of informality, such as those of Eastern and Southern Europe; and the need to validate this scheme by adapting it to national conditions.

A crucial limitation for Latin America is the productive and occupational heterogeneity, characterized by conditions between wage earners and non-wage earners. Rose and Harrison (2010) found this tension for European cases and posed it as a problem of internal comparability: “It is well established that self-employed workers, or to be more precise, small entrepreneurs and the self-employed, are in a different position from employees working in the same type of occupations” (p. 278). This issue is particularly relevant in contexts with high levels of informality, where occupational titles may not reflect authority, autonomy, or job security. Espinoza examines these elements (tasks, autonomy, and informality) in Chile, demonstrating that the managerial class is frequently overvalued in services due to the pronounced heterogeneity of labor relations. He also points out that there are many self-employed people and small business owners working informally, which supports the need to compare different systems, create clearer classification rules, and use ESeC as a standard for validation (Espinoza, 2024). Even with these limitations, comparability exercises show that adapted EGP can retain analytical capacity. First, it serves as a general model to comprehend the class structure in Latin America, illustrating the relationship between class and social indicators like access to social security. Second, the comparison between Spain and Argentina indicates a consistent correlation between class and income, demonstrating the model’s applicability when tailored to national contexts (Solís et al., 2019; Chávez Molina and Alfageme, 2022).

Following these considerations, the Honduran adaptation proposed by Llopis et al. (2024) was adopted: (1) service class (people with the capacity to control administrative resources and in management positions, qualified autonomous professionals), (2) routine formal workers (salaried in routine tasks of lower hierarchy), (3) small employers and independent workers (small employers and non-salaried professionals), (4) formal salaried workers (salaried employees with a high or intermediate level of qualification), (5) salaried and self-employed workers in the informal sector (low-skilled employees in various economic sectors), (6) independent agricultural class (self-employed in agricultural activities), and (7) salaried agricultural class (day laborers and other salaried agricultural workers).

In Honduras, this discussion also further problematized the formal/informal distinction as an analytical criterion. In terms of measurement, a productivist approach is adopted, which assumes the size of the productive unit as a criterion for differentiating the condition of informality. However, the specific threshold is defined based on the Honduran empirical reality. For this reason, and unlike studies that maintain the cut-off at 10, we set the distinction at 5 employees (Solís et al., 2016; Chávez Molina et al., 2022). The threshold of five workers is consistent with national evidence on the predominance of micro and small enterprises. Informality in Honduras is high and transversal. It is historically reproduced in small productive units (micro and small enterprises, often family-owned) that employ fewer than five workers (Del Cid and Fidel, 2002; Consejo Hondureño de la Empresa Privada (COHEP), 2017; Michel and Walker, 2019). Likewise, recent studies combine both approaches, productivist and regulationist, to observe informality, even in more industrialized productive structures such as Argentina’s (Poy, 2024). Thus, the five-worker criterion functions as an empirical rule of classification aimed at improving internal validity.

The social class variable was defined using the National Occupational Classifier (CNOH-2018), which includes the main job and a 4-digit code for different occupation types; it also used the occupational category to tell apart salaried and non-salaried workers (Llopis et al., 2024).

An analytical limitation of the study derives from the need to recode two independent variables, a decision taken to guarantee the stability of the statistical models, although it implied the loss of their internal heterogeneity. First, in the variable “ethnic group,” Garifuna and Creole were classified under “Afro-Hondurans”; the category “Indigenous Peoples” was formed by adding Tolupanes, Pech, Miskito, Nahuas, Lencas, Tawahkas, and Maya Chortís; and the remaining population was designated as “mestizo.” Second, the variable “educational level” was redefined as follows: the category “No formal education” remained constant. The classifications “Basic (1–3)” and “Basic (4–6)” were consolidated into a category designated as “Primary.” The divisions of “Basic (7–9)” and “Middle” were consolidated into “Secondary.” The “Superior” category remained constant. Finally, the two additional independent variables, sex (male and female) and geographic location (urban and rural), were extracted directly from the data provided in the EPHPM (2024).

For the implementation of multinomial logistic regressions, the reference category in the dependent variable is “Class of services.” In the vector of regressors, the reference categories were “male” for the sex variable, “mestizo” for the ethnic group variable, “urban” for the geographical area, and “superior” for the educational level. The non-interaction model follows the econometric specification 1:

$$\begin{array}{c} \ln\left(\frac{\mathrm{Pr}\left(Y_{i}=j\right)}{\mathrm{Pr}\left(Y_{i}=1\right)}\right)=\beta_{0j}+\beta_{1j}Afro_{i}+\beta_{2j}Originarito_{i}\\ +\beta_{3j}Mujer_{i}+\beta_{4j}Rural_{i}+\beta_{5j}\sin Nivel_{i}\\ +\beta_{6j}\mathrm{Pr}imaria_{i}+\beta_{7j}\sec undaria_{i}+\varepsilon_{ij} \end{array}$$ (1)

Donde $\varepsilon_{ij}∼F\left(0,\sigma_{j}^{2}\right)$ es el término de error para el individuo *i* en la ecuación de la clase *j*.

On the other hand, the model with interactions responds to Equation 2:

$$\begin{array}{c} \ln\left(\frac{\mathrm{Pr}\left(Y_{i}=j\right)}{\mathrm{Pr}\left(Y_{i}=1\right)}\right)=\beta_{0j}+\beta_{1j}Afro_{i}+\beta_{2j}Originarito_{i}\\ +\beta_{3j}Mujer_{i}+\beta_{4j}(Afro_{i}\times Originarito_{i})\\ +\beta_{5j}(Originarito_{i}\times Mujer_{i})\\ +\beta_{6j}Rural_{i}+\beta_{7j}\mathrm{Sin}Nivel_{i}\\ +\beta_{8j}\mathrm{Pr}imaria_{i}+\beta_{9j}\sec undaria_{i}+\varepsilon_{ij} \end{array}$$ (2)

In both regressions, robust standard errors were used to control for the possible presence of heteroskedasticity by means of the vce(robust) command. All estimates were made with Stata19 software.

## 3 Results

### 3.1 Descriptive statistics

Of the total employed population in Honduras (3,724,971), Afro-Hondurans represent 0.88%, indigenous peoples 5.61%, and mestizos 93.51%. Table 1 reports the distribution of demographic and territorial characteristics by ethnic group. Afro-Hondurans are more concentrated in urban areas and have a relatively high participation in primary and secondary education. Conversely, Indigenous peoples are more concentrated in rural areas and show higher proportions at the primary education level. There are also differences in the type of activities in which the employed population participates. Among those who identify as Afro-Hondurans, 69.31% work in service activities. In contrast, among indigenous peoples, this proportion is 48.34%. Agricultural, livestock, or fishing work accounts for 33.55% of the employed indigenous population, compared with 6.73% in the case of Afro-Hondurans. On the other hand, industrial and construction activities (which do not represent class categories in themselves but help to understand labor insertion) group together 23.9% of Afro-Hondurans and 18.11% of indigenous peoples. Tables 2, 3 present descriptive statistics by ethnic group for selected sociodemographic characteristics (sex, geographic distribution, and education), along with the distribution of these characteristics across the seven macroclasses.

**Table 2:** Descriptive of ethnic groups.

Sociodemographic characteristic	Ethnic group		
	Afro-Hondurans	Indigenous peoples	Mestizos
Sex			
Men	41.1%	44.9%	45.5%
Women	58.9%	55.1%	54.5%
	100.0%	100.0%	100.0%
Geographical area			
Urban	65.4%	26.4%	56.3%
Rural	34.6%	73.6%	43.7%
	100.0%	100.0%	100.0%
Educational level			
No formal education	4.1%	8.6%	7.9%
Primary	68.9%	73.9%	71.0%
Secondary	22.4%	15.3%	17.9%
Superior	4.6%	2.2%	3.2%
	100.0%	100.0%	100.0%

Source: authors’ elaboration based on EPHPM (2024).

**Table 3:** Descriptive of social class and its distribution within ethnic groups.

Social classes recess	Afro-Hondurans	%	Indigenous peoples	%	Mestizo	%	Total	%
Class of services	4,498	19.73	21,603	9.89	421,649	12.95	447,750	12.81
Routine Formal Workers	1,004	4.40	5,462	2.50	190,478	5.85	196,944	5.63
Small employers and freelancers	7,473	32.78	56,449	25.85	853,108	26.21	917,030	26.23
Salaried formal workers	3,188	13.98	18,031	8.26	493,473	15.16	514,692	14.72
Salaried and self-employed workers in the informal sector	4,408	19.34	38,010	17.40	630,974	19.38	673,392	19.26
Self-employed workers in the agricultural sector	1,116	4.90	34,056	15.59	228,095	7.01	263,268	7.53
Salaried workers in the agricultural sector and unpaid family members in agricultural activities	1,109	4.86	44,796	20.51	437,243	13.43	483,147	13.82
Total	22,797	100	218,406	100	3,255,020	100	3,496,223	100

Source: own elaboration based on EPHPM (2024).

### 3.2 Multinomial model without interactions

To estimate the econometric models, three robustness checks were carried out. First, the basic model without interactions was compared with the model including interactions; the latter showed a better fit (ΔLL = 5,453.2, 12 df, *p* < 0.001), justifying the inclusion of the ethnicity × sex interaction term. Second, the coefficients of the control variables in both models exhibited stability. Third, as indicated in the Methods section, the models were estimated with robust standard errors to control for potential heteroskedasticity. In addition, Hausman and McFadden (1984) tests were run for the class categories by estimating the model excluding one category at a time. The Independence of Irrelevant Alternatives assumption held in four comparisons (*p* > 0.05). A marginal violation was detected when excluding the “Small employers and self-employed workers” category (chi2 = 9.69, *p* = 0.046), concentrated on the sex effect and not on the ethnicity variables central to the study (Table 4).

**Table 4:** Parameters of the basic multinomial model.

Parameter	Value
Pseudo R	0.1639
Forest	151,840.18***

Source: authors’ elaboration based on the results of the multinomial logit model. Robust standard errors.

The Afro-Honduran coefficient between the full model and the specification that excludes that category remained stable (from −1.394 to −1.384, *p* < 0.01 in both cases). In addition, quasi-perfect separation was identified for the Afro-Honduran × Woman interaction in the informal self-employed category, which would reflect the reduced presence of Afro-Honduran women in this segment.

Table 5 presents the estimated associations between ethnic group and class position in the multinomial model. Afro-Hondurans show higher predicted probabilities of being classified within the service sector (4.7%) and among employers and independent workers (5.0%) relative to the reference category; in addition, the data indicate negative values for agricultural categories.

**Table 5:** Predicted probabilities of social class by ethnic group.

Variable/category	Class I	Class II	Class III	Class IV	Class V	Class VI	Class VII
Ethnic group							
Afro-Honduran	0.047	−0.019	0.050	−0.023	0.022	−0.024	−0.053*
Indigenous peoples	0.012	−0.023*	0.004	−0.044*	0.002	0.041*	0.006
Sex							
Women	0.027*	0.016*	0.223*	−0.095*	0.020*	−0.066*	−0.126*
Geographical area							
Rural	−0.073*	−0.057*	−0.028*	−0.129*	−0.022*	0.109*	0.200*
Educational level							
No formal education	−0.601*	−0.091*	0.108*	0.037	0.241*	0.101*	0.205*
Primary	−0.550*	−0.038*	0.119*	0.123*	0.167*	0.055*	0.124*
Secondary	−0.307*	0.007	0.032	0.090*	0.096*	0.020	0.062*

Source: authors’ elaboration based on the results of the multinomial logit model. Importance: ***s < 0.01, **s < 0.05, *s < 0.10.

Afro-Hondurans show higher predicted probabilities in lower-skilled service categories and lower predicted probabilities in managerial service positions (see Annex 1). Similarly, in the categories of small employers and self-employed workers, Afro-Hondurans within the ‘small employers and independent workers’ macroclass, predicted probabilities are higher in self-employed positions than in employer positions (Annex 1).

In contrast, indigenous peoples are significantly less likely to be in classes of service (1.2%) or among employers and freelancers (0.4%); however, their probabilities of belonging are higher in the independent agricultural classes (4.1%), as well as in those of salaried workers and unpaid family members in the same sector (0.6%). In addition, they show greater probabilities of insertion in informal salaried positions. Indigenous peoples show higher predicted probabilities in independent agricultural classes and informal salaried positions.

After adjusting for sex, geographical area, and educational level, the magnitude of several ethnic differences is reduced. For example, Afro-Hondurans show a lower probability of belonging to agricultural classes (−5.3%) after controlling for sex, geographical area, and educational level.

Similarly, indigenous peoples are less likely to enter formal occupations (−4.4%), even with similar levels of education. For example, women, by their sex, are more likely to be in service classes (2.7%), as well as in non-manual or independent occupations (2.3%). On the other hand, they are less likely to be inserted into formal (−9.5%) or agricultural (−12.6%) salaried classes. Women show higher predicted probabilities of belonging to service classes (2.7%) and non-manual or independent occupations (2.3%), and lower probabilities of insertion in formal salaried (−9.5%) and agricultural classes (−12.6%).

The estimates show differences by geographical area, particularly between rural and urban population. For example, people living in rural areas are less likely to be included in classes associated with the service sector (−7.3%) and formal wage earners (−12.9%). However, the probability of being part of independent agricultural classes is 10.9%. Similarly, salaried workers and unpaid family workers in this sector make up 20%.

Educational level is associated with differences in predicted probabilities across class positions. Individuals without formal education show lower predicted probabilities in the service class (−60%) and higher predicted probabilities in agricultural and informal classes (20%). Individuals with secondary education show higher predicted probabilities in formal salaried and service classes. Higher educational attainment is associated with higher predicted probabilities in service and formal salaried positions. Model 2 includes an interaction term between ethnicity and sex. Table 6 presents the corresponding predicted probabilities. The data show that women are more likely than men to belong to the service class, especially Afro-Hondurans (10.1%), followed by women from indigenous peoples (6.4%) and mestizos (2.4%).

**Table 6:** Parameters of the multinomial model with the interaction between ethnic group and sex.

Parameter	Value
Pseudo R	0.1644
Forest	165,056.06***

Source: authors’ elaboration based on the results of the multinomial logit model.

Women from indigenous and mestizo groups show predicted probabilities of 28.1 and 22.1%, respectively, in the ‘small employers and self-employed’ class (Table 6).

The probability of belonging to agricultural macroclasses is lower for women across all ethnic groups, particularly among Afro-Hondurans (−5.9%), Indigenous peoples (−16.4%), and mestizos (−12.4%). In contrast, men show higher predicted probabilities of belonging to these agricultural macroclasses. The interaction estimates show differentiated patterns by sex within each ethnic group, particularly in service, self-employed, and agricultural class positions (see Table 7).

**Table 7:** Mean marginal effects of sex on the probability of belonging to each social class, by ethnic group.

Class	Afro-Hondurans	Indigenous people	Mestizo
Class I	0.101	0.064*	0.024*
Class II	0.011	0.029	0.015*
Class III	0.098	0.281*	0.221*
Class IV	−0.005	−0.095*	−0.096*
Class V	−0.100	−0.007	0.022*
Class VI	−0.045	−0.108*	−0.063*
Class VII	−0.059	−0.164*	−0.124*

Source: authors’ elaboration based on the results of the multinomial logit model. Importance: ***s < 0.01, **s < 0.05, *s < 0.10.

### 3.3 Multinomial model with interactions

The second model was estimated including an interaction between ethnicity and sex. The likelihood-ratio test shows that the interaction model has a statistically significant better fit (*p* < 0.001). The pseudo R2 increased from 0.1639 to 0.1644 and the Wald statistic rose from 151,840.18 to 165,056.06 (see Table 6).

The ethnicity × sex interaction was estimated controlling for educational level and geographic area. Women have higher predicted probabilities than men of belonging to the service class: Afro-Honduran women 10.1%, Indigenous women 6.4%, and mestizo women 2.4%. Mestizo women constitute the largest share of the employed female population. Differences for Afro-Honduran and Indigenous women are reported as above.

Indigenous and mestizo women have predicted probabilities of 28.1 and 22.1%, respectively, for membership in the small-employers and self-employed class (skilled or semi-skilled) (see Table 7).

Predicted probabilities of belonging to the agricultural macroclasses are lower for women: Afro-Honduran women −5.9%, Indigenous women −16.4%, and mestizo women −12.4%. Underreporting of rural women’s work is noted. Mestizo and Indigenous men have higher representation in agricultural classes, as reported in the estimates.

In summary, the interaction results report that sex and ethnicity are associated with differences in class position; these associations are presented together with geographic location and educational level.

## 4 Discussion

### 4.1 Intersectionality of inequalities: race, gender, education and territory

The patterns reported in the Results section can now be interpreted in light of broader structural inequalities related to ethnicity, gender, education, and territory. The results of the analysis show that, in Honduras, it is important to relate class structure to ethnicity, gender, education, and territory. These dimensions intersect and reinforce each other. None of these variables alone can explain social positions; rather, their effects depend on how they interact within the social and economic system (Rose and Harrison, 2007; Solís and Boado, 2016). The descriptive distributions already show differentiated patterns of sectoral insertion. Afro-Hondurans are more represented in-service activities and industrial segments, whereas indigenous peoples are more concentrated in agricultural sectors. These differences anticipate the interaction between ethnicity and territory discussed below.

The results presented propose that the effects of the association between ethnicity and class position vary according to access to education and geographical location. Higher levels of schooling and urbanization concentrate the Afro-Honduran population in service classes and urban self-employment. Indigenous peoples, who have limited access to education and a significant rural presence, reside mainly in agricultural or informal sectors (Rivas, 1994; World Bank, 2015; Michel and Walker, 2019). The reduction in ethnic differences observed after controlling for educational attainment and geographical area suggests that part of the stratification patterns is structured through spatial and educational inequalities rather than ethnicity alone. This pattern indicates that work is divided according to ethnicity and location, as documented in studies on historical inequalities in rural development in Honduras (Palacios, 2007; Euraque, 2023).

The observed social disparities appear to be closely linked to education and territoriality. Although education increases the likelihood of accessing formally salaried positions, its impact is limited by factors such as rurality and continued disparities in access to and quality of education (World Bank, 2024; ECLAC, 2017; Solís et al., 2019). Rural regions, which are home to a significant portion of the indigenous population, face substantial structural barriers to educational and labor mobility (Velásquez, 2021).

The exclusion of indigenous and Afro-Honduran populations, as well as women, from access to formal and well-paid jobs cannot be attributed solely to their ethnic origin or gender. It is critical to examine the interaction of these factors with education and geographic location. This finding aligns with the traditional concept of intersectionality (Crenshaw, 1991). This concept holds that social hierarchies arise from interrelated systems of power, such as racism, capitalism, and patriarchy. Its effects are not additive; rather, they are interconnected, producing distinct forms of inequality and exclusion within contexts.

### 4.2 Education and social classes

The results highlight the relevance of the educational variable, especially considering the high levels of informality found in the study. However, having advanced educational degrees does not guarantee direct entry into the service sector, as this process is affected by other factors of exclusion or division of jobs (Rose and Harrison, 2007; Solís and Boado, 2016).

The findings suggest that the influence of the educational dimension varies according to the geographical, rural–urban location. Solís et al. (2016) and Solís et al. (2019) argue that territory and education play a crucial role in the formation of social position, educational performance, and job opportunities. Individuals residing in rural regions with limited educational attainment encounter challenges in pursuing further studies. This phenomenon is evident among indigenous and mestizo populations in these regions, where restricted educational opportunities compel individuals to pursue less formalized agricultural roles and occupations.

In contrast to the statements of Solís and Boado (2016), Pérez Sáinz (2014), and Telles and Martínez Casas (2019) about the intersectional study of class structure that encompasses education, territory, gender, and ethnicity, the Honduran case offers a nuanced perspective. In this perspective, some variables (especially education) appear consistently associated with class position, particularly education, in the formation of social classes. The interaction between education and territory indicates that occupation depends on the credentials of individuals and the disposition of the productive structures available for their integration.

ECLAC (2017), ILO (2024), and Gontero and Vezza (2023) emphasize how gender and education are connected, noting that more women are taking urban jobs and that there are differences in job performance, mainly due to the way work is divided by gender, and the results show that women, especially those from indigenous and Afro-Honduran communities, work mainly in the urban self-employment and service sectors. Despite having superior educational trajectories compared to men, women have limited access to formal education.

### 4.3 The relevance of rurality in relation to social class

Among the dimensions analyzed, education and territory show strong associations with class position. By defining classes based on the jobs held by individuals or families (Solís et al., 2016), this study finds a marked association between geographical area and class position in the place that a person occupies in the social structure. In rural areas, for example, the independent agricultural class and agricultural wage earners and unpaid family members predominate. The magnitude of the differences observed between rural and urban populations suggests that territorial location plays a structuring role in the Honduran class system.

Similar findings were made by Del Cid (2012), who found that self-employed people and unpaid family members are the most common occupational categories in rural areas, followed by salaried workers. The low level of diversification of the rural economy is one of its characteristics; it is still mostly agrarian and inefficient in production, which aggravates structural inequality. According to Llopis et al. (2024), production in the agricultural sector remains traditional, as evidenced by the creation of goods with minimal value added.

This phenomenon reflects a characteristic of Latin American countries (Chávez Molina et al., 2022): differences in their class structures. The countries can be classified into three groups: the countries of the Southern Cone (Argentina, Chile, and Uruguay), which have a high proportion of their population in the service class and the routine non-manual class; Brazil and Mexico, which share processes of early industrialization with the former group, are more diverse in terms of racial and ethnic composition, and they have a significant rural population that affects their class structures. A third group of countries includes Ecuador, El Salvador, Nicaragua, and Peru, which are characterized by lower levels of urbanization and industrialization, as well as the importance of primary economic activities.

In the third group, similar to Honduras, rural classes remain a significant component of the class structure (Chávez Molina et al., 2022). This reflects the fact that Latin American societies remain rigid in their patterns of social mobility (Solís et al., 2016). To illustrate this, at the regional level, indigenous people living in rural areas have fewer opportunities to complete education at all levels compared to their urban counterparts (World Bank, 2015).

### 4.4 Gender dynamics within ethnic groups

Gender intersects with inequalities linked to ethnicity, education, and territory. The observed patterns show that gender plays an important role in the organization of social classes, affecting how different ethnic-racial groups integrate into the labor market and access different types of employment and social protection.

Education and geographical location shape the association between ethnicity and social class, while gender inequalities aggravate these differences. Men are mostly concentrated in formal salaried classes and in the agricultural sector, while women (particularly Afro-Honduran and indigenous women) are more likely to be inserted in the service sector and in urban self-employment. The division of occupations aligns with broader trends, where a greater number of women engage in informal work, often characterized by lower pay and less social protection. In the Honduran context, Velásquez (2021) documents that women face greater difficulties in accessing the labor market, which translates into more fragmented occupational trajectories than those of men.

The results, viewed from an intersectional perspective, suggest that the distribution of gender inequalities among ethnoracial groups is not uniform. Afro-Honduran women tend to be inserted into non-manual occupations or autonomous activities in urban environments, while indigenous women are concentrated in agricultural, domestic, or subsistence work, which is often under-recorded in labor statistics (Rivas, 1994; Palacios, 2007). The lower participation of women in agricultural macroclasses observed in the models may also reflect the documented underreporting of rural women’s work in labor statistics (Rivas, 1994; Palacios, 2007). This difference is consistent with previous evidence showing that rural women have a constant lag in access to productive resources and economic opportunities compared to urban women (Velásquez, 2021). In this sense, the precarious situation experienced by Afro-descendant women in cities and the lack of variety in the rural work of indigenous women are contexts that help us understand the patterns that are observed (Euraque, 2023; ECLAC, 2017).

It is also important to consider the compositional structure of the employed population when interpreting these differences. Mestizo women constitute the largest share of employed women in the sample, which may influence the magnitude of some estimated probabilities. Therefore, differences observed between Afro-Honduran, Indigenous, and mestizo women should be interpreted in light of this demographic distribution.

While the empirical analysis focuses on labor insertion and class positions, these results should be read in light of a broader social environment marked by persistent gender inequalities. Previous studies have documented that, even as women’s participation in formal employment increases, many women access precarious or low-paid jobs and face structural barriers to fully exploiting their productive time (Velásquez, 2021). The literature indicates that the models employed in this study do not directly reflect the dimensions of gender inequality in Honduras. These include unequal access to social security and greater female exposure to different forms of structural violence, including sexual crimes and domestic violence (Velásquez, 2021). These contextual conditions do not constitute empirical findings of the present analysis, but they are relevant to understanding the structural limits of women’s economic autonomy. The magnitude and consistency of the sex differences across class categories suggest that gender operates as a structuring axis of class allocation in Honduras, shaping how ethnic groups are distributed within the labor market.

The results support what ECLAC (2020) calls “conditional economic autonomy”: women are in the labor market, but they do so in precarious conditions, with little variety of jobs and with little participation in important decisions. This configuration contributes to the reproduction of differentiated patterns of labor integration and social mobility in the Honduran context.

### 4.5 Implications for social stratification and public policy

From a social stratification perspective, the results suggest that policies that address education, employment, gender, or ethnicity in isolation tend to be insufficient to modify existing class hierarchies. This finding is important for creating public policy because it indicates that actions that focus only on increasing education or getting formal work do not change the systems that maintain inequality.

In this sense, the empirical results allow us to problematize the scope of the existing policy instruments in Honduras. The Public Policy against Racism and Racial Discrimination for the Integral Development of Indigenous and Afro-Honduran Peoples (P-PIAH) explicitly recognizes the structural nature of racism and racial discrimination and adopts principles of human rights, gender equality, and interculturality (Secretaría de Desarrollo e Inclusión Social (SEDIS), 2015). However, the patterns seen in this study indicate that, in reality, government actions have not been very effective in changing the specific ways that job separation and job insecurity impact Indigenous and Afro-Honduran women differently.

The evidence presented indicates that the number of women in low-productivity jobs and unprotected jobs is directly related to two issues addressed by P-PIAH: the right to decent work and access to ancestral heritage resources. However, the results show that the lack of coordination between employment policies, intercultural education policies, and territorial development strategies has reduced the impact of these initiatives on the class structure. This finding matches what P-PIAH has diagnosed, showing that many indigenous and Afro-Honduran people still face high poverty, informal jobs, and economic exclusion, even though there are many laws in place (Secretaría de Desarrollo e Inclusión Social (SEDIS), 2015).

Likewise, the findings related to territorial differentiation reinforce the need to strengthen the territorial approach of public policies. The study reveals that in rural areas, indigenous women face greater challenges due to their social class, limiting their ability to diversify their work and find formal jobs, while in cities, Afro-Honduran women mostly work in informal and self-employed jobs. This empirical pattern is directly connected to the strategic axes of the P-PIAH related to the right to land, territory, and natural resources, in addition to promoting community development. However, it also shows the difficulties in translating these principles into lasting improvements in the employment structure.

The results indicate that a public policy to reduce inequality in Honduras needs to move from sectoral approaches to interventions that address at the same time the problems of labor segmentation, inequality in education, differences between regions, and racial and gender discrimination. In this framework, the affirmative action measures contemplated in the P-PIAH acquire relevance not only as normative instruments. They can also be useful for changing the social status of groups that have been left out in the past, as long as they are properly connected with job opportunities, education that respects different cultures, and local development policies (Secretaría de Desarrollo e Inclusión Social (SEDIS), 2015).

## 5 Conclusion

The application of the Erikson–Goldthorpe–Portocarrero (EGP) scheme to Honduras indicates that the class structure within the country is organized in an intersectional manner. In this context, gender, ethnicity, education, and rurality collectively influence the position of various groups within the social hierarchy, although their impacts vary.

There are differences between ethnic groups; however, its impact on class position is moderated by educational level and geographic location. Ethnicity does not solely determine an individual’s position within the social hierarchy but rather interacts with multiple structural factors that can intensify or alleviate inequalities. This evidence suggests that, although each factor is significant, none alone explains belonging to a particular social class. The effect depends on interactions with variables such as educational level and geographic territory.

People with a low level of education and who live in rural areas are less likely to join formal or service classes. The situation worsens for Afro-Honduran and indigenous women, who face greater obstacles to accessing formal employment, even when they reach educational levels comparable to those of men. On the other hand, groups with a higher level of education and residents of urban areas are better able to access service classes and the formal sector, which could generally translate into better living conditions. These could show that there is still a division of labor, where gender and ethnicity act as factors limiting employment opportunities for some and increasing them for others.

Honduras establishes the territory as a fundamental element in its social structure. The rural area continues to be a context in which historical inequalities are perpetuated, derived from limited productive diversification and the lack of formal employment opportunities. As a result, people living in these regions are often employed in agricultural sectors, either as independent or salaried workers, as well as in unpaid family work, a phenomenon recognized in previous research (Llopis et al., 2024). This pattern suggests that rurality functions as a geographical dimension and a structural axis of social inequality in Honduras.

In summary, the attempt to adapt the EGP scheme to the reality of Honduras resulted in an analytical tool that can be used to study social classes in relation to gender and ethnic-racial identity. However, we found no central factor to explain the position of indigenous and Afro-Honduran peoples in the country’s current social structure. The identification of the weight of gender, educational level, and territory in class belonging made it possible to highlight the particular forms of inequality experienced by these groups in the labor market. As noted above, recognizing how these factors converge to create social inequalities is a critical step in developing better public policies that address these challenges in all their complexity.

## Data Availability

The raw data supporting the conclusions of this article will be made available by the authors, without undue reservation.
