# Supplementary material for: Ethnicity, gender, and social class in Honduras: an analysis using the permanent household survey
Source: Front Sociol. 2026 May 26;11:1758677. doi: 10.3389/fsoc.2026.1758677 (PMC13246388; doi:10.3389/fsoc.2026.1758677)
Supplement: Supplementary file 1 [file Table_1.DOCX]

Supplementary Material

# Supplementary Figures and Tables

**Table 1. Class structure and ethnic group. Source: Authors**

| **Macro class** | **Social class (15)** | **Afro descendants** | **Indigenous people** | **Mestizos** |
| --- | --- | --- | --- | --- |
| **Class of services** | Professionals, administrators, and senior officials, managers of large establishments | 0.6% | 1.2% | 1.7% |
|  | Professionals or self-employed professionals | 1.1% | 0.8% | 2.9% |
|  | Professionals, administrators, and junior officials | 17.2% | 6.9% | 8.3% |
| **Regular formal workers** | High-level non-manual routine employees (administration and commerce) | 3.3% | 1.3% | 3.6% |
|  | Non-manual routine employees, sales in large companies (more than 5 employees) | 2.2% | 1.5% | 3.3% |
| **Small employers and self-employed workers** | Employers with fewer than 5 employees | 3.9% | 2.5% | 2.8% |
|  | Self-employed in skilled and semi-skilled occupations | 26.7% | 21.2% | 22.3% |
| **Formal salaried workers** | Manual, skilled, and semi-skilled workers in large establishments (more than 5 employees) | 15.6% | 7.6% | 12.9% |
|  | Manual, unskilled workers in large establishments (more than 5 employees) | 3.3% | 3.2% | 4.9% |
| **Salaried and self-employed workers in the informal sector** | Employees in small retail establishments (fewer than 5 employees) | 1.7% | 2.5% | 2.4% |
|  | Self-employed in unskilled occupations | 5.6% | 3.6% | 3.2% |
|  | Manual, unskilled, and semi-skilled workers in small establishments (fewer than 5 employees) | 6.1% | 5.4% | 6.7% |
|  | Manual, unskilled workers in small establishments (fewer than 5 employees) | 5.5% | 6.3% | 6.3% |
| **Independent agricultural class** | Self-employed workers in the agricultural sector | 2.2% | 15.6% | 6.3% |
| **Salaried agricultural workers** | Salaried workers and unpaid family members in agricultural activities | 5.0% | 20.4% | 12.4% |
| **Total** | | 100.0% | 100.0% | 100.0% |
